# Pre‐eclampsia risk‐stratified planned birth at term: A survey of women’s perspectives on acceptability and risk communication

**DOI:** 10.1111/aogs.70230

**Published:** 2026-05-25

**Authors:** James Goadsby, Angel Leung, Sergio A. Silverio, Laura A. Magee, Peter von Dadelszen, Argyro Syngelaki, Kypros H. Nicolaides, Kayleigh S. Sheen

**Affiliations:** ^1^ Harris Birthright Research Centre for Fetal Medicine King’s College Hospital London UK; ^2^ Department of Women & Children’s Health, School of Life Course & Population Health Sciences King’s College London London UK; ^3^ Department of Psychology, Institute of Population Health University of Liverpool Liverpool UK; ^4^ School of Social Sciences, College of Health, Science and Society University of the West of England Bristol UK

**Keywords:** acceptability, patient surveys, pre‐eclampsia, risk communication, trial evaluation

## Abstract

**Introduction:**

Pre‐eclampsia (PE) remains a major complication of pregnancies, globally, and while there is no intervention proven to be effective in decreasing late preterm and term PE; the PREVENT‐PE trial aimed to evaluate whether the intervention—a strategy of screening for PE risk at 35–36 weeks' gestation, and for a risk of ≥1‐in‐50, offering risk‐stratified, planned early term birth—could reduce the incidence of subsequent PE, compared with usual care.

**Material and Methods:**

We aimed to evaluate the acceptability of trial participation and risk communication for women participating in The PREVENT‐PE trial [ISRCTN41632964] in the United Kingdom. From March to October 2024, trial participants were invited, antenatally and/or postnatally, to complete an online survey, and acceptability and risk communication measures were compared by trial arm.

**Results:**

Of those accessing the survey, 257 out of 478 (48.2%) were antenatal (median 35.4 weeks' gestation), and 485 out of 1617 (30.0%) were postnatal (median 25.6 weeks postnatally) respondents who were eligible. Antenatally, and by trial arm: (i) most women felt: comfortable with the trial (>70%); participation required little/no effort (>75%; 77.9% [intervention] vs. 91.7% [control], *p* = 0.05); confident in participation (>75%); the trial was acceptable (≈90%); and PE risk screening was fair (>90%), could reduce chances of becoming seriously ill (>90%), and understood how that could be achieved (>90%); (ii) half disagreed that timed birth for PE would interfere with competing priorities, although 20–30% agreed; (iii) ≥80% of participants felt listened to, were able to ask questions and receive helpful answers, had their preferences considered, had clear on the situation being discussed, and were confident about their decision; and (iv) ≈50% were uncertain about what was going to happen. Postnatally, views were more neutral, without an increase in negativity.

**Conclusions:**

Findings indicate that women find third‐trimester screening for PE and risk‐stratified planned early term birth highly acceptable, understand those risks, and the potential to favorably impact their pregnancy outcome.

## Graphical abstract

The survey was designed to assess participants' acceptability of the PREVENT‐PE trial, with survey questions adapted to address each acceptability construct: affective attitude (comfort with involvement); burden (effort taken for involvement); ethicality (screening is fair for all); perceived effectiveness (perceptions of likely benefit); intervention coherence (ability to reduce risk); self‐efficacy (confident of doing what is required); opportunity costs (impact on other priorities); and overall acceptability.

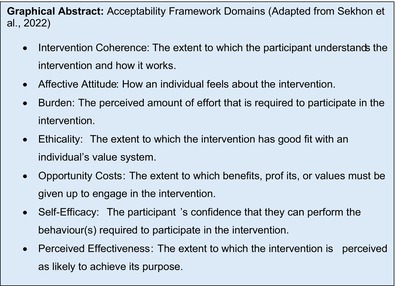

**Key Message**

Third‐trimester screening for pre‐eclampsia risk, and risk‐stratified personalized timed birth at term was found to be acceptable to women.

## 1 INTRODUCTION

Pre‐eclampsia (PE) complicates up to 8% of pregnancies, globally.^1^ Screening for PE risk is most accurately undertaken by the Fetal Medicine Foundation (FMF) competing‐risks model at 11–13 weeks' gestation, when those with a risk of at least 1% are offered aspirin, which reduces the risk of preterm PE by ≈60%,^2^ but does not affect the incidence of term PE. Assessment of late preterm and term PE risk is most accurately undertaken by the FMF competing‐risks model at 35^+0^ to 36^+6^ weeks' gestation, when ≈70% of women at risk can be identified. Whilst there is no intervention proven to be effective in decreasing late preterm and term PE, a randomized trial of low‐risk nulliparous women showed that planned early term birth at 39^+0–4^ weeks' gestation significantly reduced development of gestational hypertension or PE.^3^

The PREVENT‐PE trial^4^ aimed to evaluate whether the intervention—a strategy of screening for PE risk at 35–36 weeks' gestation, and for a risk of ≥1‐in‐50, offering risk‐stratified, planned early term birth—could reduce the incidence of subsequent PE (the primary outcome), compared with usual care. In this open, randomized trial, 8136 unselected women who were attending a routine fetal ultrasound scan and provided written, informed consent were randomized to the intervention or usual care. The intervention (vs. usual care) was associated with a 30% reduction in the incidence of PE (158/4037, 3.9% vs. 226/4057, 5.6% respectively; adjusted risk ratio [aRR] 0.70, 95% confidence interval (CI) 0.58–0.86), with no difference in emergency Caesarean (922/4031, 22.8% vs. 878/4048, 21.6%, respectively; aRR 1.06, 95% CI 0.97–1.15) or neonatal unit admission ≥48 h (261/4031, 6.5% vs. 275/4048, 6.8%, respectively; aRR 0.96, 95% CI 0.81–1.13).^4^

To support the implementation of the PREVENT‐PE clinical trial results, information was required about the experiences of participants who received care in the trial. This is a common aspect of implementation‐evaluation studies of clinical trials.^5^ Despite, counseling about PE risk‐screening being widely recommended across international clinical practice guidelines,^6^ there is a surprising dearth of data about its acceptability to care‐users. Most of this data relate to screening for either chromosomal abnormalities in the first trimester, or fetal structural abnormalities in the second trimester.^7^, ^8^, ^9^ In pregnancy hypertension specifically, acceptability has been evaluated indirectly through women's understanding and satisfaction with two blood pressure control strategies,^10^ perceived barriers and facilitators to participation in a timing of birth trial,^11^ and reasons for retention in a trial of antihypertensive therapy for chronic or gestational hypertension.^12^ However, a comprehensive assessment of acceptability should evaluate the domains underlying this concept: how people feel about a management strategy (affective attitude); the perceived effort required for involvement in that management (burden); alignment of the management with personal values (ethicality); understandability of the process (intervention coherence); the “costs” of involvement (opportunity costs); efficaciousness of the management (perceived effectiveness); and the extent to which participants feel able to undertake any requirements of them (self‐efficacy).^13^ Also, as PREVENT‐PE involved PE risk‐screening in the intervention arm, women were counseled that if they participated, they would be informed of their absolute risk level, what it would mean for their care, and what if any, timed birth were offered. Despite the commonplace nature of risk communication in maternity care, there is little clarity in the published literature about risk communication best practice.^14^

## 2 MATERIAL AND METHODS

The results of the PREVENT‐PE Trial (prospectively registered: ISRCTN41632964) have been published^4^ and the results are summarized above. The trial was approved by the National Health Service Health Research Authority London—Surrey Research Ethics Committee (reference 22/LO/0794) and all study participants provided written, informed consent. The trial was registered (ISRCTN41632964, 1 Nov 2022) prior to recruitment.

The PREVENT‐PE protocol^15^ included an implementation‐evaluation component, consisting of patient and staff surveys and interviews, approved on 15 June 2024 by the NHS Health Research Authority London—Surrey Research Ethics Committee (reference 22/LO/0794) via an amendment. Through structured antenatal and postnatal surveys of trial participants, this study sought to assess the acceptability of PREVENT‐PE trial participation and the views of participants on the quality of the PE risk communication received. This manuscript focuses on the survey results regarding the views of women on trial acceptability and risk communication.

To be reported separately are survey results reflecting staff views on trial acceptability and PE risk communication, the impact of trial participation on women's mental health, and qualitative analysis of the interviews with women and staff (including women who declined participation).

The online survey was hosted by Qualtrics and was open for completion by trial participants from 6 March to 22 October 2024. Following recruitment to the PREVENT‐PE trial, women were asked to complete the antenatal survey. If so willing, they received brief instructions and a QR code with their unique trial identification number which linked to the online survey. They entered their identification number into the survey and received an online information sheet and consent form to sign. The instructions explained that women could answer all questions, even if they were in the usual care group, because they had been willing to be in either trial arm before randomization. To facilitate antenatal survey completion, women were offered the use of an electronic tablet device within the hospital setting, or alternatively, they could complete the survey on their own device at any point before birth. To capture the views of women after birth, a link to an online postnatal survey was sent by email to all participants in the trial to date. Each survey took approximately 5–7 min to complete.

Women were eligible to be included if they consented, confirmed their trial participant status (intervention or usual care), and for this analysis, completed data for acceptability or risk communication measures.

Part 1 of the survey was designed to assess participants' acceptability of the PREVENT‐PE trial, with survey questions adapted from Sekhon et al.^16^ to address each acceptability construct: affective attitude (comfort with involvement), burden (effort taken for involvement), ethicality (screening is fair for all), perceived effectiveness (perceptions of likely benefit), intervention coherence (ability to reduce risk), self‐efficacy (confident of doing what is required), opportunity costs (impact on other priorities), and overall acceptability.^16^ Each item was tailored to refer specifically to the PREVENT‐PE trial and PE risk screening. Response options were presented on a 5‐point Likert scale, with higher scores representing a more positive endorsement of the item. These items were identical on antenatal and postpartum surveys, which are provided in Appendix S1.

Part 2 of the survey contained questions assessing participants' satisfaction with, and the impact of, risk‐related consultation in PREVENT‐PE. Given the absence of a standardized tool to assess risk communication in a maternity setting, questions were adapted from existing literature.^17^ Items assessed participants' perceptions of how well they felt listened to; their ability to ask questions and receive helpful answers, whether their preferences were asked about and respected, their awareness and certainty of what was being discussed and what would happen, and confidence in their decision to participate. Nine items were developed and scored on a scale of 1 (strongly disagree) to 5 (strongly agree). A total summed score was calculated to indicate general satisfaction with risk‐related consultation. Internal consistency of the scale was excellent (antenatal: *α* = 0.82/*ω* = 0.85; postnatal: *α* = 0.93/*ω* = 0.94), meaning the total score reliably reflects a single, general construct that each item influences. The questionnaires are provided in Appendix S2.

The following data were recorded from the PREVENT‐PE trial: demographic information (e.g., age, Index of Multiple Deprivation,^18^ ethnicity, body mass index), group allocation (intervention/control), PE risk status in the intervention group, and medical and obstetric history (e.g., gestational age if pregnant or number of weeks postpartum, method of conception, smoking status, history of diabetes).

Descriptive analyses were conducted for all study variables, using SPSS Statistics, version 30. Acceptability and perceptions of risk communication were analyzed separately by antenatal and postnatal survey completion and by trial allocation group (intervention or usual care). Chi‐square testing was conducted to examine between‐group differences in categorical variables. For all inferential analyses, statistical significance was set at *p* < 0.05.

## 3 RESULTS

**FIGURE 1:**
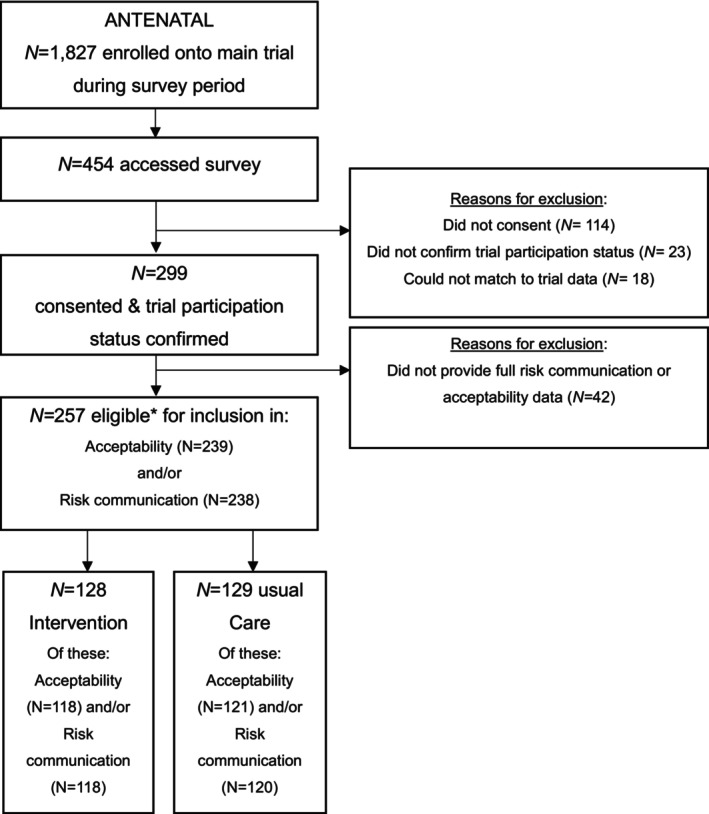
PREVENT‐PE survey for ANTENATAL women flow diagram of participation. * Eligibility was defined as women consenting to survey participation, confirming their trial participant status (as intervention or usual care), and completing data for acceptability and/or risk communication measures.

**FIGURE 2:**
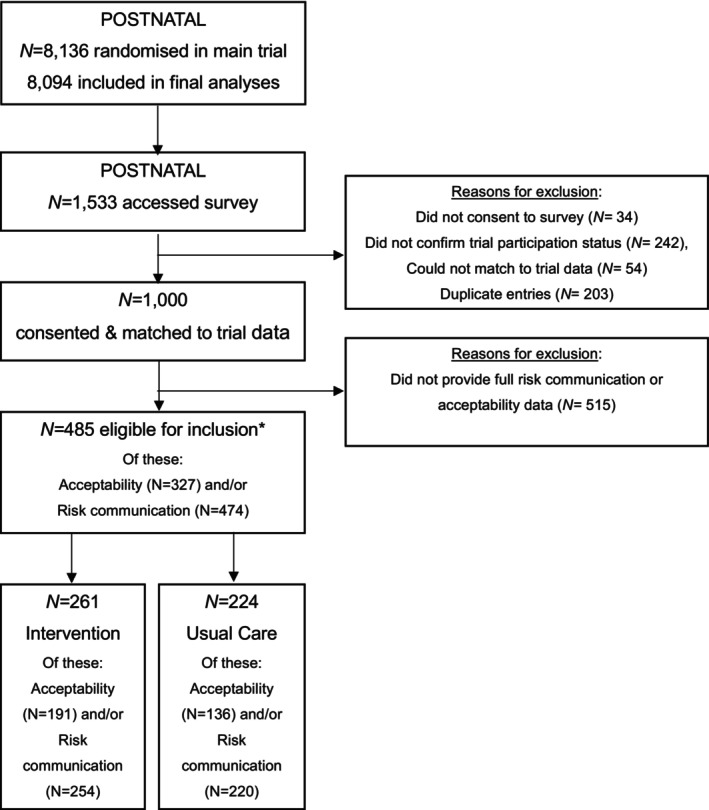
PREVENT‐PE survey for POSTNATAL women flow diagram of participation. * Eligibility was defined as women consenting to survey participation, confirming their trial participant status (as intervention or usual care), and completing data for acceptability and/or risk communication measures.

From 6 March to 22 October 2024, all women in PREVENT‐PE were asked to complete the antenatal and/or postnatal survey, as applicable. One thousand eight hundred and twenty‐seven participants were recruited during this time period. By the trial end, 8136 participants had been randomized. For the antenatal survey, 257 out of 454 (56.6%) trial participants who accessed the survey were eligible (Figure 1). Women completed the survey between 35 and 40 weeks' gestation (median of 35.6 weeks, IQR of 6). For the postnatal survey, 485 out of 1533 (31.6%) women who accessed the survey were eligible (Figure 2). Women completed the survey between 1 and 69 weeks after birth (median of 24.0 weeks, IQR of 23). Completion was similar by intervention (*N =* 128/257, 49.9% antenatal, *N =* 261/485, 53.8% postnatal) and usual care (*N* = 129/257, 50.1% antenatal, *N* = 224/485, 46.2% postnatal) groups.

### 3.1 Participant characteristics

Table 1 presents participants' characteristics at trial enrolment, as well as pregnancy outcomes, by antenatal or postnatal participation, and by trial arm. Women ranged in age from 17.6 to 46.3 years (mean of 33.4 years) in the antenatal survey, and 17.8 to 50.6 years (mean of 33.9 years) in the postnatal survey. Most women were of White ethnicity, with slightly more non‐White ethnicity women among antenatal (24.8%) than postnatal (18.0%) respondents. All strata of social deprivation were represented. Few respondents were smokers and just under 10% had conceived through the use of assisted reproductive technologies. Less than 2% of women had a prior co‐morbidity, such as chronic hypertension, pre‐gestational diabetes mellitus, or antiphospholipid syndrome. A minority of women reported prior PE (3%–4%) or a prior small‐for‐gestational‐age infant (7%–9%). These demographics were similar by trial arm, antenatal or postnatal trial participation, and similar to those of participants in the PREVENT‐PE trial overall (as previously reported^4^). Also, just under 25% of women were induced, about 22% had no labor, 7% developed PE, just over 20% gave birth by emergency Caesarean, and about 5% of babies were admitted to neonatal care for at least 48 hours, similar to the PREVENT‐PE trial overall (Tables 3).

**TABLE 1:** Maternal demographic and pregnancy characteristics of participants.

Characteristics	Total trial cohort (*N =* 8094)^4^		Antenatal survey participants (*N =* 257)						Postnatal survey participants (*N =* 485)					
			Total antenatal		Screening (*N =* 128)		Usual care (*N =* 129)		Total postpartum		Screening (*N =* 261)		Usual care (*N =* 224)	
	*N*	%	*N*	%	*N*	%	*N*	%	*N*	%	*N*	%	*N*	%
Self‐declared ethnicity														
White	5996	74.1	188	73.2	96	75.0	92	71.3	405	83.5	218	83.5	187	83.5
Black	1090	13.5	36	14.0	17	13.3	19	14.7	33	6.8	16	6.1	17	7.6
South Asian	517	6.4	15	5.8	8	6.3	7	5.4	20	4.1	12	4.6	8	3.6
More than one	312	3.9	13	5.1	5	3.9	8	6.2	15	3.1	12	4.6	3	1.3
East Asian	179	2.2	5	1.9	2	1.6	3	2.3	12	2.5	3	1.1	9	4.0
Index of multiple deprivation														
1 and 2 (most deprived)	1407	17.4	26	10.1	13	10.2	13	10.1	63	13.0	33	12.6	30	13.4
3 and 4	2093	25.9	73	28.4	33	25.8	40	31.0	129	26.6	70	26.8	59	26.3
5 and 6	1841	22.7	59	23.0	31	24.2	28	21.7	116	23.9	69	26.4	47	21.0
7 and 8	1600	19.8	65	25.3	34	26.6	31	24.0	103	21.2	54	20.7	49	21.9
9 and 10 (least deprived)	1153	14.2	34	13.2	17	13.3	17	13.2	74	15.3	35	13.4	39	17.4
Smoking	221	2.7	8	3.1	5	3.9	3	2.3	4	0.8	0	0	4	1.8
In vitro fertilization or ovulation induction	537	6.6	18	7.0	11	8.6	7	5.5	41	8.4	22	8.4	19	8.5
Diabetes mellitus														
Type 1	26	0.3	1	0.4	1	0.8	0	0	4	0.8	3	1.1	1	0.4
Type 2	40	0.5	1	0.4	0	0	1	0.8	2	0.4	0	0	2	0.9
Systemic lupus erythematosus	24	0.3	0	0	0	0	0	0	1	0.2	1	0.4	0	0
Antiphospholipid syndrome	18	0.2	1	0.4	1	0.8	0	0	1	0.2	0	0	1	0.4
Chronic hypertension	73	0.9	5	1.9	2	1.6	3	2.5	4	0.8	3	1.1	1	0.4
Parity														
Nulliparous			137	53.3	68	53.1	69	53.5	229	47.2	127	48.7	102	45.5
Previous history of preeclampsia	251	3.1	10	3.9	4	3.1	6	4.7	14	2.9	8	3.1	6	2.7
Previous history of a small‐for‐gestational age infant	675	8.3	25	9.7	14	10.9	11	8.5	34	7.0	19	7.3	15	6.7
Onset of labor														
Spontaneous	NA	NA	NA	NA	NA	NA	NA	NA	258	53.2	136	52.1	122	54.5
Induction of labor	NA	NA	NA	NA	NA	NA	NA	NA	118	24.3	64	24.5	54	24.1
No labor	NA	NA	NA	NA	NA	NA	NA	NA	108	22.3	61	23.4	47	21.0
Missing	NA	NA	NA	NA	NA	NA	NA	NA	1	0.2	0	0	1	0.4
Primary outcome of trial														
Preeclampsia									34	7.0	11	4.2	23	10.3
Key secondary outcomes of trial														
Birth by emergency Caesarean	NA	NA	NA	NA	NA	NA	NA	NA	108	22.3	63	24.1	45	20.1
Neonatal care unit admission for ≥48 h									22	4.5	9	3.4	13	5.8

### 3.2 Acceptability of trial participation

Overall, perceived acceptability was high among antenatal (mean ± SD score of 4.64 ± 0.71 out of a potential score of 5) and postnatal respondents (mean ± SD of 4.17 ± 0.99 out of 5). Table 2 shows that the vast majority of respondents regarded PREVENT‐PE as highly favorable, with positive perceptions of both the practicalities of the trial protocol and principles of antenatal risk screening.

**TABLE 2:** Participant views of acceptability of PREVENT‐PE trial participation and preeclampsia screening, by antenatal and postnatal survey and trial allocation.

Question	Response	Antenatal (*N =* 239)				*p*	Postnatal (*N =* 327)				*p*
		Intervention (*N =* 118)		Usual care (*N =* 121)			Intervention (*N =* 191)		Usual care (*N =* 136)		
		*N*	%	*N*	%		*N*	%	*N*	%	
How comfortable did you feel being part of the PREVENT trial	Very comfortable	61	51.7	61	50.4	0.963	69	36.1	44	32.4	0.506
	Somewhat comfortable	26	22.0	27	22.3		36	18.8	23	16.9	
	Neither comfortable nor uncomfortable	6	5.1	9	7.4		40	20.9	34	25.0	
	Somewhat uncomfortable	2	1.7	2	1.7		16	8.4	7	5.1	
	Very uncomfortable	23	19.5	22	18.2		30	15.7	28	20.6	
How much effort did it take to be involved in the PREVENT Trial?	No effort at all	51	43.2	61	50.4	0.054	96	50.3	82	60.3	0.090
	A little effort	41	34.7	50	41.3		53	27.7	38	27.9	
	A moderate amount	22	18.6	8	6.6		32	16.8	12	8.8	
	A lot of effort	1	0.8	1	0.8		6	3.1	4	2.9	
	A huge amount	3	2.5	1	0.8		4	2.1	0	0.0	
How confident did you feel that you could do what is required to be involved in the PREVENT trial?	Very confident	69	58.5	68	56.2	0.784	74	38.7	54	39.7	0.667
	Somewhat confident	26	22.0	21	22.3		39	20.4	29	21.3	
	Neutral	23	19.5	25	20.7		69	36.1	49	36.0	
	Not very confident	0	0	0	0		6	3.1	1	0.7	
	Not confident at all	0	0	1	0.8		3	1.6	3	2.2	
How acceptable was the PREVENT trial for you?	Completely acceptable	86	72.9	93	76.9	0.437	95	49.7	72	52.9	0.279
	Somewhat acceptable	23	19.5	15	12.4		44	23.0	23	16.9	
	Neither acceptable nor unacceptable	8	6.8	12	9.9		42	22.0	38	27.9	
	Somewhat unacceptable	0	0	0	0		6	3.1	1	0.7	
	Completely unacceptable	1	0.8	1	0.8		4	2.1	2	0.6	
Please indicate the extent you agree with: “Receiving screening for pre‐eclampsia risk is fair for all”	Strongly agree	90	76.3	98	81.0	0.297	143	74.9	95	69.9	0.864
	Somewhat agree	19	16.1	17.0	14.0		32	16.8	26	19.1	
	Neither agree not disagree	8	6.8	3	2.5		10	5.2	10	7.4	
	Somewhat disagree	0	0	0	0		3	1.6	3	2.2	
	Strongly disagree	1	0.8	3	2.5		3	1.6	2	1.5	
Please indicate the extent to which you agree with; “Screening for pre‐eclampsia is likely to improve my chances of not becoming seriously ill”	Strongly agree	94	79.7	97	80.2	0.946	137	71.7	103	75.7	0.658
	Somewhat agree	17	14.4	16	13.2		39	20.4	20	14.7	
	Neither agree nor disagree	6	5.1	6	5.0		10	5.2	7	5.1	
	Somewhat disagree	0	0	0	0		2	1.0	2	1.5	
	Strongly disagree	1	0.8	2	1.7		3	1.6	4	2.9	
Please indicate the extent to which you agree with; “It is clear to me how screening for pre‐eclampsia risk will reduce my risk of becoming severely ill”	Strongly agree	92	78.0	99	81.8	0.667	132	69.1	91	66.9	0.974
	Somewhat agree	20	16.9	15	12.4		40	20.9	29	21.3	
	Neither agree nor disagree	5	4.2	4	3.3		7	3.7	5	3.7	
	Somewhat disagree	1	0.8	2	1.7		7	3.7	7	5.1	
	Strongly disagree	0	0	1	0.8		5	2.6	4	2.9	
Please indicate the extent to which you agree with; “Timed birth for pre‐eclampsia interferes with my other priorities”	Strongly disagree	36	30.5	42	34.7	0.385	57	29.8	48	35.3	0.458
	Somewhat disagree	16	13.6	21	17.4		24	12.6	20	14.7	
	Neither agree nor disagree	29	24.6	33	27.3		64	33.5	45	33.1	
	Somewhat agree	13	11.0	11	9.1		29	15.2	12	8.8	
	Strongly agree	24	20.3	14	11.6		17	8.9	11	8.1	

Amongst antenatal respondents in both the intervention and usual care arms, most women felt comfortable with the trial protocol (>70%) and felt that participation required little or no effort (>75%) (Table 2). Whilst there was a significant minority of women who felt very uncomfortable with the trial protocol in both antenatal and postnatal groups (both just under 20%, with no apparent increase in the intervention [vs. usual care] group), this negative sentiment was not reflected in subsequent responses. There were also numerically fewer women in the intervention (vs. usual care) group who endorsed that the trial required no or little effort (77.9% vs. 91.7%, respectively; *p* = 0.05). Most women were confident in their participation (>75%) and felt that the trial was acceptable (≈90%, with >70% overall ranking it as “completely acceptable”). Most participants agreed that PE risk screening was fair for all (>90%) and likely to improve the chances of not becoming seriously ill (>90%), with most understanding how that could be achieved (>90%). Whether timed birth for PE would interfere with their other priorities did not differ between trial groups, but a minority of participants (20–30%) agreed with this statement.

Amongst postnatal respondents, there were more neutral (rather than positive) responses to the acceptability questions, but there was no swing seen from positive to negative perceptions of acceptability (Table 2). As for antenatal respondents, numerically fewer women in the intervention (vs. usual care) group endorsed that the trial required no or little effort (78.0% vs. 88.2%, respectively; *p* = 0.09).

### 3.3 Risk perception

Table 3 shows that antenatally and postnatally, regardless of trial allocation arm, the risk communication strategies used in the trial were viewed positively (and usually very positively) by the vast majority of respondents. There were no items in which there was a majority of unfavorable responses.

**TABLE 3:** Participant views of risk communication in the PREVENT‐PE trial, by antenatal and postnatal survey and trial allocation.

		Antenatal (*N =* 238)					Postnatal (*N =* 474)				
		Intervention (*N =* 118)		Usual care (*N =* 120)			Intervention (*N =* 254)		Usual care (*N =* 220)		
Question: Thinking about the information you received about the PREVENT trial, please indicate the extent to which you agree with the statements…											
		*N*	%	*N*	%	*p*	*N*	%	*N*	%	*p*
Overall, I felt listened to	Strongly agree	74	62.7	81	67.5	0.645	80	31.5	67	30.5	0.751
	Somewhat agree	26	22.0	22	18.3		72	28.3	60	27.3	
	Neither agree nor disagree	15	12.7	16	13.3		87	34.3	76	34.5	
	Somewhat disagree	0	0	0	0		5	2.0	9	4.1	
	Strongly disagree	3	2.5	1	0.8		10	3.9	8	3.6	
My concerns were listened to	Strongly agree	74	62.7	75	62.5	0.917	79	31.1	67	30.5	0.981
	Somewhat agree	21	17.8	23	19.2		65	25.6	55	25.0	
	Neither agree or disagree	22	18.6	20	16.7		93	36.6	80	36.4	
	Somewhat disagree	0	0	0	0		9	3.5	10	4.5	
	Strongly disagree	1	0.8	2	1.7		8	3.1	8	3.1	
I was able to ask questions	Strongly agree	101	85.6	99	82.5	0.534	107	42.1	95	43.2	0.499
	Somewhat agree	12	10.2	11	9.2		76	29.9	51	23.2	
	Neither agree nor disagree	5	4.2	9	7.5		58	22.8	59	26.8	
	Somewhat disagree	0	0	0	0		6	2.4	7	3.2	
	Strongly disagree	0	0	1	0.8		7	2.8	8	3.6	
The answers to my questions were helpful	Strongly agree	88	74.6	82	68.3	0.587	90	35.4	76	34.5	0.418
	Somewhat agree	18	15.3	22	18.3		74	29.1	51	23.2	
	Neither agree nor disagree	12	10.2	15	12.5		74	29.1	78	35.5	
	Somewhat disagree	0	0	0	0		10	3.9	7	3.2	
	Strongly disagree	0	0	1	0.8		6	2.4	8	3.6	
My preferences were asked about	Strongly agree	81	68.6	77	64.2	0.680	75	29.5	75	34.1	0.153
	Somewhat agree	16	13.6	18	15.0		71	28.0	51	23.2	
	Neither agree nor disagree	19	16.1	21	17.5		75	29.5	74	33.6	
	Somewhat disagree	2	1.7	2	1.7		24	9.4	10	4.5	
	Strongly disagree	0	0	2	1.7		9	3.5	10	4.5	
My preferences were respected	Strongly agree	82	69.5	87	72.5	0.598	82	32.3	81	36.8	0.229
	Somewhat agree	17	14.4	12	10.0		74	29.1	49	22.3	
	Neither agree nor disagree	19	16.1	20	16.7		78	30.7	78	35.5	
	Somewhat disagree	0	0.0	0	0.0		12	4.7	5	2.3	
	Strongly disagree	0	0.0	1	0.8		8	3.1	7	3.2	
I felt aware of the situation being discussed	Strongly agree	78	66.1	83	69.2	0.718	90	35.4	82	37.3	0.091
	Somewhat agree	30	25.4	27	22.5		83	32.7	52	23.6	
	Neither agree nor disagree	10	8.5	9	7.5		60	23.6	67	30.5	
	Somewhat disagree	0	0	0	0		14	5.5	8	3.6	
	Strongly disagree	0	0.0	1	0.8		7	2.8	11	5.0	
I felt uncertain of what was going to happen	Strongly agree	22	18.6	16	13.3	0.273	54	21.3	44	20.0	0.736
	Somewhat agree	16	13.6	9	7.5		50	19.7	41	18.6	
	Neither agree nor disagree	17	14.4	23	19.2		76	29.9	78	35.5	
	Somewhat disagree	20	16.9	18	15.0		45	17.7	32	14.5	
	Strongly disagree	43	36.4	54	45.0		29	11.4	25	11.4	
I felt confident about my decision	Strongly agree	79	66.9	81	67.5	0.623	88	34.6	81	36.8	0.318
	Somewhat agree	26	22.0	23	19.2		73	28.7	47	21.4	
	Neither agree nor disagree	12	10.2	16	13.3		78	30.7	82	37.3	
	Strongly disagree	1	0.8	0	0.0		8	3.1	6	2.7	

Antenatally, at least 80% of participants, in each trial arm, felt that they (and their concerns) had been listened to, that they were able to ask questions and the answers were helpful, their preferences had been explored and respected, that they were aware of the situation being discussed, and confident about their decision (Table 3). Only for *“I felt uncertain of what was going to happen”* did just over 50% of respondents agree, but similarly in intervention and usual care groups (i.e., “strongly disagree” in 36.4% and 45.0%, respectively; and “somewhat disagree” in 16.9% and 15.0%, respectively).

Postnatally, the results were similarly positive for the majority of respondents (Table 3). However, there was a small tendency among all responses to move toward neutrality, without a shift towards an overall negative response on any domain.

In a *post hoc* sensitivity analysis, estimated marginal means for trial acceptability (Table S1) and risk perception (Table S2) were evaluated for each trial group, adjusted for time since birth and with the respective ANCOVA annotation. Time since birth was not significant (all *p* values >0.05).

## 4 DISCUSSION

In our survey of PREVENT‐PE trial participants, we observed that the PREVENT‐PE trial was highly acceptable to respondents across all surveyed domains. Women understood the purpose and benefits of screening and felt that risk communication was appropriate. Acceptability was similar in both trial arms, indicating that women felt screening was valuable, regardless of whether they had their risk communicated (which was in the intervention arm, but not in usual care). Views appeared to be more neutral the more remote the survey was completed from trial participation, with no associated increase in negative views. Of note, numerically, fewer (although still the vast majority) of women in the intervention (vs. usual care) group felt trial participation took little or no effort, and a substantial minority of women expressed some isolated discomfort with trial participation (which may have reflected poor understanding of the question, but regardless, warrants cautious interpretation). Furthermore, and again for all women, only a minority (20–30%) of respondents felt timed birth for PE would interfere with their other priorities and expressed uncertainty about “what was going to happen.”

Acceptability incorporates both cognitive and affective responses to involvement or receipt of an intervention.^13^ The extent to which an intervention is deemed acceptable can determine participation decisions, subsequent adherence to the intervention protocol, and may also influence intended outcomes or efficacy.^16^ In previously published randomized trials on the prevention or management of PE, there have been evaluations of patient experience published alongside the main trial data. However, these have consisted of single or otherwise limited metrics evaluating patient acceptability^19^, ^20^ or health economic analysis.^12^, ^21^ Our findings extend this work to give a more comprehensive view, utilizing multiple evaluation tools validated for use in health research. This multi‐faceted evaluation of how risk screening is perceived by women to whom it is offered, and the effects of communicating that risk to patients, gives a deeper understanding of how interventions can be designed to be acceptable to the greatest number of women. Responses were probed further in qualitative work to be reported separately.^15^

Efforts to enhance risk communication have involved various approaches, based on the principle of the quality of communication through: open and honest dialogue about options; having thought processes explained; structuring the delivery of information into easily understood components^17^, ^22^; communicating verbally and non‐verbally in a way that conveys a sense that the patient is being listened to and has the opportunity to ask questions and receive answers; and presenting risk information numerically, visually, or in a personalized manner.^23^, ^24^, ^25^ The latter approach has been shown to increase intervention uptake in trials, albeit without much evidence that it reduces associated anxiety.^24^ The satisfaction with risk communication was assessed using a bespoke nine‐item scale, as no other suitable existing tool could be identified in the literature, but it limits external validity and comparison with other trials.

The study benefitted from a large number of completed sets of both antenatal and postnatal data. Whilst response rates were low overall (57% antenatally and 32% postnatally), other studies have reported 62% as good postnatally.^26^ Lower response rates may be expected when acceptability assessment occurs in parallel to the main trial, rather than embedded into routine data collection. Nevertheless, responses were balanced across both trial arms, and the relatively low variance in the data is indicative of a generally positive consensus overall. Survey participants had ethnic and socio‐economic characteristics that were representative of the ethnically and socio‐economically diverse population of women recruited to the main PREVENT‐PE trial. Thus, our findings should be generalizable to third‐trimester PE risk screening in the UK maternity population.

With respect to limitations, the social science aspect of PREVENT‐PE was added after the main trial started; given rapid recruitment, antenatal survey respondents could be approached only in the last 4 months of the trial, limiting recruitment numbers. There were a number of sources of potential selection bias. First, due to time constraints, surveys were produced only in English, thereby limiting access to women without reasonable English proficiency. Second, for antenatal or postnatal survey responses, participants with stronger trial engagement or especially positive or negative trial experiences may have been more likely to respond to the survey, affecting trial acceptability estimates. Third, many postnatal surveys were completed by participants at least 6 months after birth, which may have blunted more strongly held opinions if they existed. Fourth, the majority of women in this trial had been offered multivariable first trimester screening for PE risk (similar to the third trimester risk assessment offered in PREVENT‐PE), alongside screening for chromosomal abnormalities; while PE clinical risk factor screening is routine antenatal practice globally, and PE multivariable risk assessment was routine practice at the study sites, prior experiences of screening in pregnancy (whether positive or negative) may have influenced perceptions of screening which were then reflected in the uptake of screening in PREVENT‐PE. Finally, there were methodological issues. Women entered their own study identification number into the Qualtrics survey software, and errors meant that online survey responses (18 antenatal and 52 postnatal; Figures 1 and 2) could not be matched for some respondents. Satisfaction with risk communication was assessed using a bespoke nine‐item scale, as no other suitable existing tool could be identified, but exploration is required of external validity.

Finally, we recognize that by delaying the start of the social science evaluation, we were unable to capture the experiences of all women who participated in the trial and future trials should embed social science evaluation—for women and healthcare professionals—from the commencement to the end of trial recruitment while also giving consideration to longitudinal (antenatal to postnatal) outcomes. Future studies should also attempt to replicate this work outside of a free‐at‐point‐of‐use healthcare system and pay particular attention to strategies that would further enhance the number of ethnic minority participants, such as surveys produced in multiple foreign languages. Also, further work should explore how best to support women's general uncertainty about the birth process.

## 5 CONCLUSION

Our findings suggest that third‐trimester screening for PE risk, and risk‐stratified personalized timed birth at term is acceptable to women, who, when counseled, they come to understand their risks and how they may be addressed.

## AUTHOR CONTRIBUTIONS

JG: acquired the necessary funding, coordinated data collection, participated in the interpretation of data, wrote the first draft of the manuscript, and accessed and verified the underlying data reported in the manuscript. AL: coordinated data collection, participated in the interpretation of data, and accessed and verified the underlying data reported in the manuscript. KSS: informed methods of data collection, analyzed the data, participated in the interpretation of data. SAS: informed methods of data collection, analyzed the data, participated in the interpretation of data. LAM: conceived and designed the study, participated in the interpretation of data, wrote the first draft of the manuscript. PvD: conceived and designed the study, participated in the interpretation of data. AS: conceived and designed the study, supervised the conduct of the study, participated in the interpretation of data, accessed and verified the underlying data reported in the manuscript. KHN: conceived and designed the study, supervised the conduct of the study, participated in the interpretation of data. All authors edited the manuscript and approved the final version for submission.

## FUNDING INFORMATION

The study was supported by a grant from the Fetal Medicine Foundation (UK Charity No: 1037116). Reagents and equipment for the measurement of serum PlGF and sFlt‐1 were provided free of charge by Thermo Fisher Scientific.

## CONFLICT OF INTEREST STATEMENT

All authors declare that they have no known competing financial interests or personal relationships that could have influenced the work reported. All authors confirm that they had full access to all the data in the study and accept responsibility for submitting it for publication.

## ETHICS STATEMENT

The results of the PREVENT‐PE Trial (prospectively registered: ISRCTN41632964) have been published and summarized above. The trial was approved by the National Health Service Health Research Authority London—Surrey Research Ethics Committee on April 5, 2023 (reference 22/LO/0794) and all study participants provided written, informed consent. The trial was registered (ISRCTN41632964, November 1, 2022) prior to recruitment.

## Supporting information


Data S1.

## Data Availability

The datasets generated and/or analyzed during this study are not publicly available due to their sensitive and potentially identifiable nature, but a de‐identified dataset may be available from the corresponding author (KHN) upon reasonable request, with input from the co‐investigator group where applicable, and in accordance with the data sharing policies of King’s College Hospital.
