# Supplementary material for: Pre‐eclampsia risk‐stratified planned birth at term: A survey of women’s perspectives on acceptability and risk communication
Source: Acta Obstet Gynecol Scand. 2026 May 25;105(8):1553–65. doi: 10.1111/aogs.70230 (PMC13356484; doi:10.1111/aogs.70230)
Supplement: Supplementary file 1 — Data S1. [file AOGS-105-1553-s001.docx]

**SUPPLEMENTARY APPENDIX**

| Document | Title | Page number |
| --- | --- | --- |
| Appendix 1 | PREVENT-PE questionnaire for women (Part 1) | 24 |
| Appendix 2 | PREVENT-PE questionnaire for women (Part 2) | 24 |
| Supplementary Table S1 | Estimated marginal means for trial allocation and acceptability items, adjusted for number of weeks postpartum at time of survey completion | 26 |
| Supplementary Table S2 | Estimated marginal means (EMM) for trial allocation and risk perception, adjusted for number of weeks postpartum at time of survey completion | 27 |

**APPENDIX 1**: PREVENT-PE questionnaire for women (Part 1)

| **Acceptability of the PREVENT trial** | |
| --- | --- |
| 1. | How comfortable did you feel to [be involved in the PREVENT Trial] |
| 2. | How much effort did it take to be involved I the PREVENT trial? |
| 3. | Receiving [timed birth for pre-eclampsia risk] is fair to all |
| 4. | There are ethical consequences associated with [timed birth for pre-eclampsia risk] |
| 5. | [Timed birth for pre-eclampsia risk] Is likely to improve my chances of not becoming seriously ill |
| 6. | It is clear to me how [timed birth] will reduce my risk of becoming severely ill from pre-eclampsia |
| 7. | How confident do you feel that you can do what is required to [be involved in the PREVENT Trial?] |
| 8. | Timed birth for pre-eclampsia risk interferes with my other priorities |
| 9. | How acceptable was the PREVENT trial for you? |

**APPENDIX 2**: PREVENT-PE questionnaire for women (Part 2)

| **Satisfaction and impact of risk-related consultations** | |
| --- | --- |
| 1. | Overall, I felt listened to |
| 2. | My concerns were listened to |
| 3. | I was able to ask questions |
| 4. | The answers to my questions were helpful |
| 5. | My preferences were asked about |
| 6. | My preferences were respected |
| 7. | I felt aware the situation being discussed |
| 8. | I felt uncertain of what was going to happen |
| 9. | I felt confident about my decision |

| **Supplementary Table S1**: Estimated marginal means (EMM) for trial allocation and acceptability items, adjusted for number of weeks postpartum at time of survey completion* | | | | | | | | | | |
| --- | --- | --- | --- | --- | --- | --- | --- | --- | --- | --- |
|  |  | *EMM* | *SE* | 95% Confidence Interval | | *F* | *df_1_* | *df_2_* | *p* | Partial ᶯ^2^ † |
|  |  |  |  | Lower | Upper |  |  |  |  |  |
| How comfortable did you feel to be part of the PREVENT trial? | Screening | 3.50 | .11 | 3.292 | 3.715 | .553 | 1 | 318 | .458 | .002 |
|  | Usual care | 3.38 | .13 | 3.127 | 3.630 |  |  |  |  |  |
| How much effort did it take to be involved in the PREVENT trial? | Screening | 1.77 | .07 | 1.646 | 1.901 | .478 | 1 | 318 | .120 | .007 |
|  | Usual care | 1.54 | .08 | 1.386 | 1.689 |  |  |  |  |  |
| How confident did you feel that you can do what is required to be involved in the PREVENT trial? | Screening | 3.91 | .07 | 3.767 | 4.055 | .251 | 1 | 318 | .617 | .001 |
|  | Usual care | 3.97 | .09 | 3.797 | 4.139 |  |  |  |  |  |
| How acceptable was the PREVENT trial for you? | Screening | 4.15 | .07 | 4.003 | 4.288 | .311 | 1 | 318 | .577 | .001 |
|  | Usual care | 4.21 | .09 | 4.039 | 4.377 |  |  |  |  |  |
| Receiving screening for pre-eclampsia risk is fair for all | Screening | 4.63 | .06 | 4.520 | 4.748 | 1.250 | 1 | 318 | .264 | .004 |
|  | Usual care | 4.53 | .07 | 4.397 | 4.669 |  |  |  |  |  |
| Screening for pre-eclampsia risk is likely to improve my chances of not becoming seriously ill | Screening | 4.61 | .06 | 4.495 | 4.725 | .020 | 1 | 318 | .887 | <.001 |
|  | Usual care | 4.60 | .07 | 4.460 | 4.734 |  |  |  |  |  |
| It is clear to me how screening for pre-eclampsia risk will reduce my risk of becoming severely ill | Screening | 4.52 | .07 | 4.384 | 4.654 | .585 | 1 | 318 | .453 | .002 |
|  | Usual care | 4.43 | .08 | 4.279 | 4.599 |  |  |  |  |  |
| Timed birth for pre-eclampsia risk interferes with my other priorities | Screening | 2.59 | .09 | 2.408 | 2.776 | 2.121 | 1 | 318 | .146 | .007 |
|  | Usual care | 2.38 | .11 | 2.162 | 2.599 |  |  |  |  |  |
| ** For some participants, the number of weeks postpartum was not recorded (N= 11), and their data were excluded from this analysis.*  *† Partial η² (eta-squared) is a measure of effect size that quantifies how much of variance in an outcome is explained by a specific factor, after accounting for other factors in the model.* | | | | | | | | | | |

| **Supplementary Table S2**: Estimated marginal means (EMM) for trial allocation and risk perception, adjusted for number of weeks postpartum at time of survey completion | | | | | | | | | | |
| --- | --- | --- | --- | --- | --- | --- | --- | --- | --- | --- |
|  |  | *EMM* | *SE* | 95% Confidence Interval | | *F* | *df_1_* | *df_2_* | *p* | Partial ᶯ^2^ |
|  |  |  |  | Lower | Upper |  |  |  |  |  |
| Overall, I felt listened to | Screening | 3.82 | .065 | 3.687 | 3.944 | .132 | 1 | 460 | .716 | <.001 |
|  | Usual care | 3.78 | .071 | 3.641 | 3.919 |  |  |  |  |  |
| My concerns were listened to | Screening | 3.78 | .066 | 3.651 | 3.909 | .091 | 1 | 460 | .763 | <.001 |
|  | Usual care | 3.75 | .071 | 3.611 | 3.891 |  |  |  |  |  |
| I was able to ask questions | Screening | 4.07 | .065 | 3.940 | 4.196 | .370 | 1 | 460 | .543 | .001 |
|  | Usual care | 4.01 | .071 | 3.871 | 4.148 |  |  |  |  |  |
| The answers to my questions were helpful | Screening | 3.92 | .065 | 3.792 | 4.047 | .839 | 1 | 460 | .360 | .002 |
|  | Usual care | 3.83 | .070 | 3.693 | 3.970 |  |  |  |  |  |
| My preferences were asked about | Screening | 3.71 | .070 | 3.568 | 3.841 | .552 | 1 | 460 | .416 | .001 |
|  | Usual care | 3.79 | .075 | 3.640 | 3.936 |  |  |  |  |  |
| My preferences were respected | Screening | 3.83 | .066 | 3.698 | 3.958 | .316 | 1 | 460 | .575 | .001 |
|  | Usual care | 3.88 | .071 | 3.742 | 4.023 |  |  |  |  |  |
| I felt aware of the situation being discussed | Screening | 3.92 | .068 | 3.791 | 4.056 | .414 | 1 | 460 | .520 | .001 |
|  | Usual care | 3.86 | .073 | 3.716 | 4.003 |  |  |  |  |  |
| I felt uncertain of what was going to happen | Screening | 3.22 | .080 | 3.059 | 3.374 | .002 | 1 | 460 | .961 | <.001 |
|  | Usual care | 3.21 | .087 | 3.040 | 3.381 |  |  |  |  |  |
| I felt confident about my decision | Screening | 3.90 | .064 | 3.770 | 4.023 | .000 | 1 | 460 | .999 | <.001 |
|  | Usual care | 3.90 | .070 | 3.760 | 4.033 |  |  |  |  |  |

** For some participants, the number of weeks postpartum was not recorded (N= 11), and their data were excluded from this analysis.*

*† Partial η² (eta-squared) is a measure of effect size that quantifies how much of variance in an outcome is explained by a specific factor, after accounting for other factors in the model.*
